# Community Health Center Efficiency. The Impact of Organization Design and Local Context: The Case of Indonesia

**DOI:** 10.34172/ijhpm.2021.19

**Published:** 2021-04-13

**Authors:** Suwatin Miharti, Rafael Wittek, Bart Los, Liesbet Heyse

**Affiliations:** ^1^Department of Competency Development and Evaluation for Policy Analyst, Center of Policy Analyst Reinforcement, The National Institute of Public Administration, Jakarta, Indonesia.; ^2^Department of Sociology, Faculty of Behavioral and Social Sciences, University of Groningen, Groningen, The Netherlands.; ^3^Department of Global Economics & Management, Faculty of Economics and Business, University of Groningen, Groningen, The Netherlands.

**Keywords:** Efficiency, Community Health Centres, Context-Design Performance, Indonesia

## Abstract

**Background:** The decentralization of the Indonesian healthcare system, launched in the year 2000, allowed the authorities of local community health centers (CHCs) to tailor their services to the needs of their clients. Many observers see this as an opportunity to increase CHC efficiency. Building on the Context Design Performance Framework, this paper assesses the extent to which efficiency variations between CHCs can be explained by the degree of fit between their organizational design characteristics and aspects of the communities in which they are embedded.

**Methods:** Data envelopment analysis (DEA) was applied to construct a measure of CHC efficiency for a sample of 598 CHCs in 2011, drawn from a publicly available Ministry of Health (MoH) dataset. Tobit regression analysis was applied to assess the impact of organization design and community characteristics and their interplay on efficiency.

**Results:** Large variations in CHC efficiency were discovered, suggesting that not all CHCs are equally capable of finding the optimal design to operate most efficiently. A significant inverted U-shape relationship was found for the organization design-efficiency link: efficiency is highest for CHCs with 1-2 horizontal units and decreases for CHCs exceeding or not reaching this number. No significant association was found between community characteristics (proportion of poor people, remote location of CHC) and CHC efficiency.

**Conclusion:** Organizational design matters for CHC efficiency, but no evidence was found for the hypothesis that a better fit between community characteristics and CHC design increases efficiency. A potential reason for this might be that CHC management’s main design challenge is how to cope with the scarce availability of well-trained health personnel.

## Background

**Key Messages**

** Implications for policy makers**

- Community health center (CHC) management might consider reviewing to what degree their current level of horizontal differentiation fosters or hampers their efficiency, in particular if the CHC is located in a non-remote area or faces a high proportion of poor people in its service coverage area.
- Whereas the socio-economic status of the population in non-remote areas may directly influence CHC efficiency, choosing the right organizational design (ie, an intermediate number of horizontal units) can buffer this effect.
- For CHCs to be able to reap the full benefits of administrative decentralization, Ministry of Health (MoH) might consider labor market policies improving the supply of sufficiently trained health personnel.

** Implications for the public**

Increased local level decision-making autonomy of community health centers (CHCs) concerning key features of organizational structure can lead to improvements in the efficiency of meeting community health needs, but it may also exacerbate regional inequalities in care provision. Living in remote and/or poor areas still affects CHC efficiency and might therefore impact the perceived quality of primary healthcare.

Community health centers (CHCs) are frontline organizations in national primary healthcare systems. They have prominent tasks in providing effective, efficient, equal, accessible and affordable healthcare to local communities.^1,2^ Many countries currently invest in improving CHC capacity to improve the health of their community.^3,4^

 The decentralization of the Indonesian healthcare system was launched in 2000, and further strengthened in 2004 through higher fiscal transfers of the healthcare budget from central to local governments.^5^ It also increased CHCs autonomy to decide on organizational function, strategy and design,^6^ and the introduction of health insurance for the poor.^7^ This transfer of resources and authority to local governments and CHCs was balanced by a mechanism of multi-layered decision space in which the Ministry of Health (MoH) retained some influence by defining minimum standards regarding the functions, organization design, and performance of CHCs.^6^

 Their increased decision space allows CHCs to tailor their services and resources to the specific needs of local communities, and to experiment with innovative solutions.^8^ This should lead to more efficient healthcare provision.

 Building on the context-design-performance (CDP) framework developed for the health sector,^3,9^ we ask to what degree there is variation in the efficiency of CHCs in Indonesia’s decentralized healthcare system, and if so, how this variation can be explained. We argue that large variations in CHC efficiency are likely and that they can be explained by differences in the fit between CHC organization design and its context. The reason is that the increased decision-space may result in suboptimal design and service-provision choices.

 Being among the first systematic quantitative assessments of CHC efficiency in Indonesia, this study may benefit policy-makers in their evidence-based efforts to improve primary healthcare organizations, particularly CHCs. Previous studies on Indonesian CHC efficiency were more exploratory in nature and covered CHCs in a particular district.^10-13^ We extend this research with a systematic comparison of CHC efficiency and their antecedents across districts.

###  Indonesia’s Healthcare System and CHCs’ Characteristics

**Figure 1:**
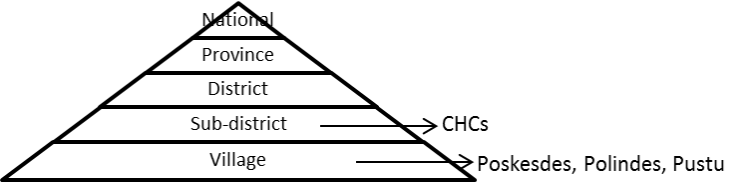
The Structure of Health Institutions in Indonesia. Abbreviation: CHCs, community health centers.

 CHCs are government health institutions at the sub-district level in Indonesia (see Figure 1). In 2011, Indonesia had 9321 CHCs, spread over 6773 sub-districts^14^. Besides CHCs, district, private and public organizations may provide healthcare to communities. To reach communities at the village level, CHCs are allowed to open branches (*Pustu ),* in each village. CHCs can request mobility facilities (ie, boats and motorcycles) to transport health staff to reach remote areas, and take the initiative to establish two other types of branches at the village level: *Polindes,* providing pre-natal and maternity care, and *Poskesdes,* providing primary care.

 Variation in functions and organization structure of CHCs is allowed as long as they meet the requirements of the MoH. The government stipulates that each CHC should employ at least eight different kinds of staff: one or more physicians, dentists, midwives, nurses, pharmacists, public health workers, nutritionists, and environmental health workers (MoH Decree no. 128/2004 on *Puskesmas*).

 CHCs can also vary in the number of horizontal units or departmentation for example, inpatient care facilities (CHCs with beds), a 24-hour facility for obstetrics neonatal care (called *Poned*), and/or an ambulatory service. The MoH determines the requirements for additional functions to be granted. For example, a CHC may have inpatient care if the sub-district is far from the hospital.

###  Hypotheses

 Being rooted in contingency theory,^15^ the CDP framework^3^ proposes three antecedents of organizational efficiency: contextual factors, organization design, and the fit between them. The core idea is that organizational performance is contingent on its ability to adapt its structure to contextual factors.^3^

###  Contextual Factors 

 Contextual factors in this study include poverty rates and the level of remoteness. First, high poverty levels, ie, the percentage of poor people in a service coverage area, reflect more severe health problems,^9^ with lower education levels and less healthy food consumption patterns leading to high morbidity rates and complex health challenges.^16^ Hence, compared to their counterparts in wealthier areas, CHCs in areas with a high proportion of poor people are likely to face more severe health problems. Lower efficiency levels may be the result because reaching the same outcome requires higher investments.

 Second, the impact of poverty is likely to be exacerbated in a remote area because bad infrastructures either inhibit patients to visit CHCs, or increase travel time.^17^ We thus hypothesize that:

*H1 (Context – poverty and remoteness)*: (*a*) The higher the poverty rate in a service coverage area, the lower the CHC efficiency. (*b*) The negative effect of poverty on CHC efficiency is stronger in remote areas than in non-remote areas.

###  Organization Design

 Organization design also influences CHC efficiency since it affects how organizations transform input into desired output.^3^

 Horizontal and spatial differentiations were found to affect efficiency positively^18^, negatively,^19^ or not at all.^20^ We argue that this inconsistency^21^ may be caused by a curvilinear (inverted U-shape) relationship between the two dimensions.

 First, horizontal differentiation can help an organization to perform more efficiently: by dividing tasks among various kinds of staff in different units or departments, specialized staff can focus on the tasks they are most qualified for, so that tasks can be completed more efficiently. This implies a positive, linear effect between the degree of horizontal differentiation and efficiency.^18,19^

 Second, there is evidence that managers tend to ask for more horizontal differentiation than may be needed, since having more units and people under managerial control increases their power and status.^22^ However, a high degree of horizontal differentiation can increase coordination costs among units. Hence, there might be a critical point at which the costs of coordination will exceed its benefits.^23-25^ We therefore expect that efficiency first increases with horizontal differentiation, but then decreases after it has exceeded a threshold value.

 Third, we expect the same logic to hold for the degree of spatial differentiation, ie, maintaining CHC offices in several different locations. *Spatial differentiation *is operationalized as the presence of CHC staff or offices in separate locations. In the Indonesian case, this refers to the number of *Pustu or Branch*, *Poskesdes,* and *Polindes*. Consequently, our hypothesis on organization design reads:

*H2 (Organization design: horizontal and spatial differentiation):* The effect of (*a*) horizontal differentiation and (*b*) spatial differentiation on CHC efficiency is curvilinear and has an inverted U shape.

###  The Context-Design Fit 

 One goal of public administration reforms is to make public services more responsive by granting them wider autonomy.^26^ This also holds for the Indonesian healthcare reform. CDP argues that this objective can be achieved if organization design fits well with contextual circumstances.^15,27^ CHCs are expected to adjust the degree of horizontal and spatial differentiation to the requirements of their service coverage area, ie, the remoteness level and poverty rates. This allows them to achieve the best possible fit, leading to effective and efficient healthcare provision^27,28^:

*H3a (Remoteness-spatial differentiation fit):* CHCs with high spatial differentiation operating in remote areas will be more efficient than CHCs with low spatial differentiation operating in remote areas. This will flatten the inverted U shape predicted for the relationship between spatial differentiation and CHC efficiency.

*H3b (Poverty-horizontal differentiation fit):* CHCs with high horizontal differentiation operating in poor areas will be more efficient than CHCs with low horizontal differentiation operating in poor areas. This will flatten the inverted U shape predicted for the relation between horizontal differentiation and CHC efficiency.

## Methods

###  Data and Sample

 The unit of analysis of this study are CHCs in Indonesia. Two data sources were combined to create this sample. First, CHC input and output data and context data on 37 districts’ health profiles was drawn from reports published by the Department of Health of each district in 2011 (ie, Health Profile of Tangerang Regency, accessed on April 27, 2014). Some reports were downloaded from the official MoH website, others from district websites. Second, basic data of CHCs was drawn from MoH’s official website (https://www.kemkes.go.id/). This data includes information on the number and nature of CHC health staff, horizontal, and spatial differentiation of CHCs.

 Data collection for both sources was arranged and coordinated by the MoH, and carried out by each district’s Department of Health. The MoH determined the data collection instruments, indicators, and structure of the report to ensure the uniformity for aggregating information at the provincial and national level.

 The year 2011 was chosen because it was the most recent year for which most information in these two data sources was available when the study was conducted. Though since 2005 all districts are expected to provide an annual health profile report, not all districts comply. Most of the available health profiles are from low to middle-income districts, indicating that they receive high fiscal transfers. One possible reason for this overrepresentation might be that government funding of the health sector depends on compliance to reporting requirements, with poorer districts depending more strongly on government money than richer ones.

 Data on CHC health performance in Indonesia are hard to find because of the under-developed infrastructure of information systems. This study is therefore based on sample of 598 CHCs in 2011 (6.4% of the total population of 9321 CHCs).

###  Dependent Variable: Technical Efficiency of CHCs

 As in previous CDP studies,^3^ we analyzed CHC efficiency in two stages. First, we estimated CHC efficiency using data envelopment analysis (DEA). Second, we tested our hypotheses with Tobit regression analysis, linking the estimated efficiency levels of CHCs to the predictors in these hypotheses. We now first discuss how the dependent variable was constructed.

 DEA is an analytical tool to benchmark an organization’s performance to the maximum attainable performance of similar organizations.^29-31^ The latter is estimated by applying linear programming methods to a sample of organizations that use similar inputs to produce similar outputs. One advantage of DEA is that it can deal with multiple inputs and multiple outputs. By virtue of the method, organizations (often labelled DMUs, ‘decision-making units’) are benchmarked only against the maximum performance of organizations that use the inputs and produce the outputs in roughly the same proportions.^31^ Another major advantage is that DEA can be used without information about the prices of inputs and outputs. Reliable information, particularly on the prices of outputs, is often lacking in the context of public organizations like CHCs.

**Figure 2:**
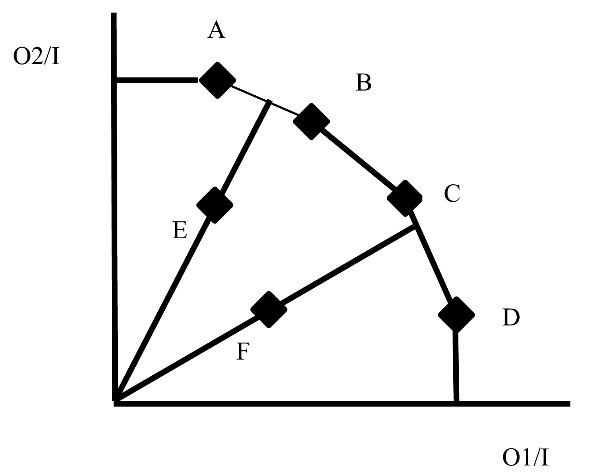
Illustration of the Estimation of Efficiency Levels in DEA. Abbreviation: DEA, data envelopment analysis.

Figure 2 illustrates how efficiency scores are determined. The example presents a context in which a single input (I) produces two outputs (O1 and O2). Six DMUs are depicted in the space that shows how much of O1 and O2 is produced with one unit of I. DMUs labelled A, B, C, and D define the ‘envelope’ or ‘frontier,’ the combinations of O1 and O2 represent maximum performance. The performances of all 6 DMUs suggest that it is not possible to produce anything more of O1 than A, B, C, and D without sacrificing some units of O2. Hence, these four DMUs on the frontier have an efficiency score of 1.

Figure 2 illustrates an ‘output-oriented’ DEA, which obtains efficiency scores by computing how much output could be expanded at given input levels. In contrast, ‘input-oriented’ DEA computes the maximum reduction in inputs for given output levels. We opt for the output-oriented alternative since we assume that CHC managements try to provide as many services as possible with given resources, rather than try to minimize resources with given service provision levels. The application of DEA analysis in this study is established by variable returns to scale, which allows considering the CHC variability in size.

*Input and output selection*. We restricted the input of the CHCs to human resources. We distinguished two groups, as in previous studies.^32^ The first group is clinical staff, who directly provide care to patients. This group consists of staff with a higher and medium educational background, physicians, dentists, and specialists, midwives and nurses. The second group is the non-clinical staff who indirectly provide care to patients. This group includes nutritionists, public health and environmental health workers, pharmacists. Since a laboratory is not a compulsory unit in a CHC, then the presence of laboratory staff is also optional. Thus, we categorize the laboratory staff as an additional profession the CHC can have, besides other health staff.

 We defined the output of CHCs based on their main goals: providing primary healthcare and mother and infant care. Mother and infant care was provided in the form of vaccination,^33^ contraception care,^33,34^ and deliveries attended by health staff.^35^ Therefore, we included the following as CHC outputs: *number of vaccinated infants,*^32^*number of active contraception users* (females of childbearing age), and *number of deliveries attended by health staff.*^32^ We also included *number of outpatient visits,*^36^ and *number of health promotion activities*, since these can help promoting breastfeeding^33,34,37^ and washing hands before feeding infants^33^ to ensure infant’s hygiene. Table 1 summarizes.

**Table 1:** Input and output Variables for Efficiency Analysis

**Variables**	**Definition**
Inputs	
Doctors	The number of physicians, dentists, and specialists
Midwives	The number of midwives
Nurses	The number of nurses
Non-clinical	The number of public health staff, nutritionists, environmental health staff, pharmacists
Laboratory staff	The number of laboratory staff
Outputs	
Vaccinated infants	The number of infants who are vaccinated
Active users of contraceptive methods	The number of couples who use contraceptive methods
Deliveries attended by health staff	The number of deliveries attended by health staff
Health promotion	The number of health promotion activities in a year
Visits	The number of outpatients visits

 CHC technical efficiency scores were estimated in two steps. First, to assess the degree of output variation, a robustness check was carried out by running DEA for different combinations of inputs and outputs. Second, to test for the presence of outliers we redid the DEA analysis involving only the non-efficient CHCs. We did not identify strong outliers.

###  Independent Variables

 Main independent variables include horizontal and spatial differentiation. *Horizontal differentiation* is operationalized by two indicators: (1) The number of different types of health staff working in a CHC, also called the *staff mix*. (2) The number of *horizontal units. *It can range from 0-3, and reflects the sum of the presence of *Poned* (24-hour care), *beds* or inpatient care, and *ambulatory service*.

*Spatial differentiation *is operationalized as the presence of CHC offices in separate locations, ie, the number of *Pustu*, *Poskesdes,* and *Polindes*.

*Contextual factors *of CHCs include the poverty rates and the level of remoteness. The *poverty* rate is operationalized as the percentage of poor people^14^ in a CHC’s coverage area.^3^ In our data, poverty rates may reach 100% since this figure also includes citizens who fall below the poverty line because one of their family members requires healthcare in clinics or hospitals. The *level of remoteness *is coded as remote (1) vs. non-remote (0) areas, based on the classification provided by the MoH.

 The remoteness level of a CHC is defined by MoH. The level of *remoteness *is categorized in two levels (remote, very remote) on the MoH website. A *remote* area is characterized by 3 main indicators: (1) its geographical position (difficult to access, disaster prone, in mountainous, inland, and swamp areas); (2) public transport is available maximally twice a week, required travelling time (return) of at least 6 hours; (3) socio-economic conditions: lack of staple goods, insecure or conflict area.

 A *very remote* area also has the following features: (1) Geographical position: tiny island, in outer or border area of the country; (2) no or no routine public transport within the area, the area can only be accessed by plane from other places, the transportation facility may be cancelled because of problematic weather conditions.

 In our analysis, we distinguish between remote and non-remote areas.

*Control Variable*. Following suggestions from previous research to avoid statistical bias, we control for *population* size in a CHC’s service coverage area.^38^ Though the MoH’s decision to create a CHC in a region is based on the population in a service coverage area reaching a certain threshold, CHCs still show some variation in the size of their service coverage areas. Since the establishment of a CHC is also related to guaranteeing accessibility to primary healthcare, CHCs are allowed to be established in areas with a population size lower than the government threshold, as is often the case in remote areas.

###  Tobit Censored Regression Analysis

 In the second stage, hypotheses were tested with Tobit censored regression, a method regularly used to analyze variation in technical efficiency.^38-40^ Tobit regression removes bias that would result from applying a standard linear regression framework to analyze truncated dependent variables (since DEA efficiency scores range from 0 to 1).^41,42^ To assess curvilinearity, we added the squares of staff mix and number of units.

## Results

###  Efficiency Analysis

Table 2 presents descriptive statistics for input and output variables. Some input indicators have zero as the minimum (ie, doctors, nurses, non-clinical, and laboratory staff), indicating that some CHCs do not meet the minimum health staff standard as determined by the MoH. Some output indicators have zero as the minimum (ie, promotion, active contraceptive users, and attended deliveries), indicating that some CHCs did not generate some core outputs.

**Table 2:** Descriptive Statistics for Input and Output Variables for Efficiency Analysis

	**Minimum**	**Maximum**	**Mean**	**Standard Deviation**
Inputs				
Doctor	0	13	2.14	1.35
Midwives	1	66	13.78	9.62
Nurses	1	45	10.51	6.41
Non-clinical	0	21	4.57	3.05
Laboratory staff	0	5	0.76	0.85
Outputs				
Infant vaccinated	33	2545	538.69	380.03
Promotion	0	5145	230.38	433.51
Active contraceptive users	0	25 982	4678.29	4133.43
Attended deliveries	0	2630	529.69	372.68
Visits	36	179 636	22 325.25	19 895.96

 We estimate CHC efficiency by analyzing the input and output indicators as described in Table 2.

Table 3 shows variation in efficiency scores. 84 (14%) of CHCs are efficient (TE score of 1). The most important finding is that as many as 364 CHCs had a TE score of less than 0.60. This variation suggests that many CHCs were not able to find a design to operate at decent levels of efficiency and that the opportunities for substantial improvements in system performance were substantial.

**Table 3:** Technical Efficiency Scores

**Efficiency Score Interval**	**No.**	**%**
0.01-0.10	20	3.34
0.11-0.20	72	12.04
0.21-0.30	83	13.88
0.31-0.40	61	10.20
0.41-0.50	67	11.20
0.51-0.60	61	10.20
0.61-0.70	41	6.86
0.71-0.80	46	7.69
0.81-0.90	37	6.19
0.91-0.99	26	4.35
1	84	14.05
*Mean 0.54*	*Total 598*	*100*

###  Descriptive Statistics 

Table 4 presents descriptive statistics, distinguishing remote from non-remote areas. The sample size with complete information on all variables is N = 355 (from N = 598).

**Table 4:** Descriptive Statistics of Independent, Dependent, and Control Variables for Tobit Regression Analysis

**Variable**	**CHC in Remote Area**					**CHC in Non-remote Area**				
	**N**	**Min**	**Max**	**Mean**	**SD**	**N**	**Min**	**Max**	**Mean**	**SD**
Independent variables										
N branch	100	0	11	3.13	2.03	320	0	9	2.39	1.51
N Polindes	91	0	27	8.74	6.00	300	0	23	6.28	4.31
N Poskesdes	85	0	8	0.62	1.18	305	0	9	0.60	1.23
N staff-mix	100	2	9	6.51	1.67	320	4	10	7.50	1.10
N horizontal unit	87	0	3	1.44	0.91	313	0	3	1.18	0.98
Poverty rates (%)	95	8.31	100	58.78	22.75	307	9.08	87.78	35.52	14.73
Dependent variables										
Efficiency score	100	0.24	1	0.53	0.22	320	0.30	1	0.79	0.17
Control variables										
Population (in thousands)	100	1.41	47.85	12.05	8.91	320	5.69	133.05	38.71	19.07
Valid N (listwise)	79					276				

Abbreviations: CHCs, community health centers; N, sample size; SD, standard deviation.

 The health staff mix ranges from 2–10, with an average of 7. This is noteworthy since the government regulation stipulated a minimal health staff mix of eight. The number of horizontal units range between 0–3, with a modus of one. The number of spatial units is dominated by the *Polindes*; 6 per CHC on average. Some CHCs may have no spatial unit. Of the n = 420 inefficient CHCs in total, 100 (23.8%) are located in remote areas.

 The total number of CHCs (observations) in the DEA analysis was 598. This number decreases to n = 420 when including remoteness and poverty rates as context variables in our Tobit analysis. We have complete data for all indicators for 79 CHCs in remote areas, and for 276 CHCs in non-remote areas, resulting in a subset of 355 CHCs for which we have complete information to carry out our Tobit analysis.

 Poverty was assessed by Central Bureau of Statistics Indonesia using an indicator based on basic needs. The *poverty* rate is calculated as the percentage of poor people in a CHC’s coverage area. Our data is based on Central Bureau of Statistics’ Social Economic and Demographic Survey 2010. Poverty rates may reach 100% since this figure also includes “nearly poor” people (the group of people that can be suddenly poor if a family member needs an intensive care hospitalization).

 The correlation between poverty and remoteness exceeds 0.6, suggesting a multicollinearity problem (Pallant, 2013). We therefore analyzed the data in two groups; one group of CHCs in non-remote areas (Table 5) and a group in remote areas (Table 6) to reduce the multicollinearity between variables and added unit squared variables in correlation analysis. As Tables 5 and 6 show, this approach solved the multicollinearity problem: all pairwise correlations are (in the majority of cases well) below 0.5, apart from those cells related to the correlations between the variables and their corresponding squared values.

**Table 5:** Correlations Between Independent Variables (CHCs in Non-remote Areas) (n = 276 CHCs)

	**Variable**	**1**	**2**	**3**	**4**	**5**	**6**	**7**	**8**	**9**	**10**	**11**	**12**
1	N Branch	1											
2	N Branch^2^	0.927^a^	1										
3	N Polindes	0.179^a^	0.150^a^	1									
4	N Polindes^2^	0.190^a^	0.172^a^	0.946^a^	1								
5	N Poskesdes	0.141^b^	0.202^a^	0.164^a^	0.140^b^	1							
6	N Poskesdes^2^	0.211^a^	0.312^a^	0.176^a^	0.182^a^	0.883^a^	1						
7	N Staff-mix	-0.062	-0.087	-0.138^b^	-0.111	0.005	0.022	1					
8	N Staff-mix^2^	-0.072	-0.093	-0.144^b^	-0.116^b^	0.000	0.016	0.992^a^	1				
9	N Horizontal Unit	0.004	0.005	0.084	0.074	0.059	0.016	0.156^b^	0.178^a^	1			
10	N Horizontal Unit^2^	0.011	0.009	0.129^b^	0.114	0.105	0.045	0.179^a^	0.202^a^	0.944^a^	1		
11	Poverty rates	0.220^a^	0.184^a^	0.215^a^	0.170^a^	0.050	0.052	-0.221^a^	-0.219^a^	-0.025	0.024	1	
12	Population	0.132^b^	0.140^b^	0.093	0.123^b^	0.317^a^	0.252^a^	-0.133^b^	-0.127^b^	0.051	0.095	-0.024	1

Abbreviation: CHCs, community health centers. ^a^Correlation is significant at the 0.01 level (Pairwise correlation). ^b^Correlation is significant at the 0.05 level (Pairwise correlation).

**Table 6:** Correlations Between Independent Variables (CHCs in Remote Areas) (n = 79 CHCs)

	**Variable**	**1**	**2**	**3**	**4**	**5**	**6**	**7**	**8**	**9**	**10**	**11**	**12**
1	N Branch	1											
2	N Branch^2^	0.927^a^	1										
3	N Polindes	0.271^a^	0.186	1									
4	N Polindes^2^	0.241^b^	0.153	0.959^a^	1								
5	N Poskesdes	0.143	0.109	0.382^a^	0.338^a^	1							
6	N Poskesdes^2^	0.184	0.176	0.231^b^	0.202	0.879^a^	1						
7	N Staff-mix	0.206^b^	0.141	0.315^a^	0.274^a^	0.098	0.103	1					
8	N Staff-mix^2^	0.212^b^	0.139	0.316^a^	0.278^a^	0.094	0.103	0.985^a^	1				
9	N Horizontal Unit	0.086	0.091	0.367^a^	0.367^a^	0.185	0.101	0.101	-0.044	1			
10	N Horizontal Unit^2^	0.036	0.032	0.373^a^	0.389^a^	0.177	0.076	0.031	0.002	0.945^a^	1		
11	Poverty rates	0.010	0.043	-0.286^a^	-0.275^b^	-0.206	-0.128	-0.233^b^	-0.247^b^	-0.164	-0.151	1	
12	Population	0.321^a^	0.282^a^	0.322^a^	0.299^a^	0.467^a^	0.408^a^	0.373^a^	0.391^a^	0.206	0.139	-0.229^b^	1

Abbreviation: CHCs, community health centers. ^a^Correlation is significant at the 0.01 level (Pairwise correlation). ^b^Correlation is significant at the 0.05 level (Pairwise correlation).

###  Tobit Regression Results

Table 7 presents the results of the Tobit regressions and contains three Models. Model A assesses the curvilinear effect of horizontal differentiation (number of horizontal units and staff mix) and organizational context (poverty). Model B adds the curvilinear interaction effect of horizontal differentiation and context (poverty) on efficiency. Model C also adds the curvilinear interaction effect of spatial differentiation.

**Table 7:** Results of Tobit Censored Regression Analysis of Technical Efficiency of CHCs

**Variable**	**Model A**				**Model B **				**Model C**			
	**Remote (n = 79)**		**Non-remote (n = 276)**		**Remote (n = 79)**		**Non-remote (n = 276)**		**Remote (n = 79)**		**Non-remote (n = 276)**	
	**Co-efficient**	**SE**	**Co-efficient**	**SE**	**Co-efficient**	**SE**	**Co-efficient**	**SE**	**Co-efficient**	**SE**	**Co-efficient**	**SE**
Context												
Poverty	0.002^a^	0.001	-0.002^b^	0.001	0.011	0.009	0.026	0.017	0.010	0.010	0.021	0.017
Horizontal Differentiation												
Units	0.063	0.053	0.044^c^	0.026	0.015	0.145	0.293^a^	0.077	0.022	0.152	0.281^a^	0.076
Units^2^	-0.020	0.017	-0.023^b^	0.009	0.004	0.045	-0.125^a^	0.027	0.008	0.050	-0.121^a^	0.027
Staff-mix	-0.361^a^	0.055	-0.233^b^	0.077	-0.318^a^	0.074	-0.230^b^	0.090	-0.327^a^	0.075	-0.225^b^	0.090
Staff-mix^2^	0.022^a^	0.005	0.015^b^	0.005	0.019	0.015	-0.002	0.012	0.021	0.016	0.000	0.013
Spatial Differentiation												
Branch	0.016	0.026	-0.011	0.016	0.009	0.025	-0.015	0.016	0.037	0.086	-0.015	0.051
Branch^2^	-0.004	0.004	0.002	0.002	-0.002	0.004	0.003	0.002	-0.011	0.014	0.010	0.009
Polindes	-0.021^b^	0.009	-0.017^b^	0.006	-0.020^b^	0.009	-0.016^b^	0.006	-0.017	0.024	-0.023	0.021
Polindes^2^	0.001^b^	0.000	0.001^c^	0.000	0.001^b^	0.000	0.001^c^	0.000	0.001	0.001	0.001	0.001
Poskesdes	-0.051	0.031	0.024	0.016	-0.047	0.030	0.029^c^	0.015	-0.088	0.094	0.063	0.050
Poskesdes^2^	0.005	0.004	-0.005^c^	0.003	0.004	0.004	-0.006^b^	0.003	0.003	0.023	-0.014	0.012
Controls												
Population	0.017^a^	0.002	0.006^a^	0.001	0.016^a^	0.002	0.006^a^	0.001	0.016^a^	0.002	0.006^a^	0.001
Interaction Effects												
Units * Poverty					0.000	0.002	-0.007^a^	0.002	0.000	0.003	-0.007^a^	0.002
Units^2^ * Poverty					0.000	0.001	0.003^a^	0.001	0.000	0.001	0.003^a^	0.001
Staff-mix * Poverty					-0.002	0.003	-0.007	0.005	-0.001	0.003	-0.005	0.005
Staff-mix^2^ * Poverty					0.000	0.000	0.000	0.000	0.000	0.000	0.000	0.000
Branch * Poverty									-0.001	0.001	0.000	0.001
Branch^2^ * Poverty									0.000	0.000	0.000	0.000
Polindes * Poverty									0.000	0.000	0.000	0.001
Polindes^2^ *Poverty									0.000	0.000	0.000	0.000
Poskesdes * Poverty									0.000	0.002	-0.001	0.001
Poskesdes^2^ * Poverty									0.000	0.001	0.000	0.000

Abbreviations: CHCs, community health centers; SE, standard error. ^a^ Correlation is significant at 0.1 level. ^b^Correlation is significant at 0.05 level. ^c^Correlation is significant at 0.01 level. n = 79 CHCs in remote areas and n = 276 CHCs in non-remote areas, N total = 355 CHCs in remote and non-remote areas.

####  Context Effects on Efficiency

 H1a predicted a negative association between poverty and efficiency, and H1b argued that this effect is stronger for remote than for non-remote areas. The analysis shows the proportion of poor people in a service coverage area does not have a direct effect on a CHC’s technical efficiency. Consequently, no evidence is found for H1a and H1b.

####  The Effects of Horizontal Differentiation on Efficiency

 H2a suggested an inverted U-shape relationship between horizontal differentiation and efficiency. Horizontal differentiation is indicated by the presence of horizontal units (horizontal units) and variation of health staff (staff mix).

 Horizontal units have a significant linear effect in Model A, but only in non-remote areas. The effects of “units” are significant and positive, the effects of “units square” are significant and negative. When the interaction effect is included in models B and C, the effect of the units remains, with higher effect sizes compared to Model A.

 According to our curvilinearity diagnostics^43,44^ (calculation available upon request), the observed parameters meet the requirements for a significant inverted U-shaped relationship between the number of horizontal units and efficiency. Thus, the findings are in line with Hypothesis H2a. We found a turning point at 1.2, suggesting that efficiency is highest in CHCs with one or two horizontal units and lower for CHCs without a horizontal unit or with more than two horizontal units.

 Staff mix, in model A, has a significant negative effect in both remote and non-remote areas, whereas the effect of staff-mix square is positive, suggesting a U-shaped effect on efficiency. In remote areas, both the effect sizes and the confidence intervals (*P* = .01) of staff mix were higher compared to those in non-remote areas (*P* = .05). When the interaction variables are included in Model B and C, the curvilinear effect of staff-mix becomes insignificant. Thus, hypothesis H2b is refuted.

####  The Effects of Spatial Differentiation on Efficiency

 H2b predicted an inverted U-shaped relationship between spatial differentiation, measured as the number of branches, *Polindes*, and *Poskesdes*. Both have significant effects in Models A and B, but these effects disappear in Model C, which contains all interaction effects. This means that spatial differentiation does not affect efficiency, neither in remote, nor in non-remote areas. Hence, no evidence could be found for H2b: there is no systematic relationship between a CHCs spatial differentiation and its technical efficiency.

####  The Effects of Poverty and Organization Design on Efficiency

 H3a argued that CHCs with high spatial differentiation operating in remote areas will be more efficient than CHCs with low spatial differentiation operating in remote areas. With none of the indicators measuring spatial differentiation, or its interaction effects having a significant effect on efficiency in Model C, H3a has to be refuted.

 According to H3b, horizontal differentiation (number of units, staff mix) pays off predominantly for CHCs operating in poor areas, resulting in poverty flattening the inverted U-shape relation between horizontal differentiation and efficiency. For the degree of staff mix, no significant interaction effects were found. As the curvilinearity statistics show (calculation available upon request), poverty indeed flattens the inverted U-shaped relationship between the number of horizontal units and technical efficiency, in line with H3b. As the results for H3b show, poverty nevertheless significantly indirectly affects CHC efficiency in non-remote areas, with horizontally highly differentiated CHCs being less efficient in poor areas than their counterparts.

####  Control Variable and Efficiency

 The control variable *population *shows significant and positive effects in all Models, suggesting that the CHCs in larger coverage areas tend to be more efficient than CHCs situated in smaller service coverage areas. However, though highly significant, effect sizes are very low.

## Discussion

 The analyses show that both organizational design and context matter for efficiency. With regard to design, horizontal differentiation has an impact, whereas none of the indicators for spatial differentiation show a systematic association with efficiency.

 In contrast, both horizontal differentiation measures affect efficiency. CHCs with a low degree of *staff mix* outperform those with a higher staff mix. This linear negative association holds for CHCs in both remote and non-remote areas, and its effect size is the second strongest in the study. The effect of staff mix holds irrespective of the three context conditions investigated here: remoteness level and poverty rates.

 Remoteness matters for the impact of *number of horizontal units*, and showing the strongest effect sizes in our study. Efficiency rates are highest for CHCs with an intermediate number (range 1–2) of horizontal units, but this effect holds only for CHCs in non-remote areas. The impact of the number of horizontal units becomes weaker when the proportion of poor people increases in non-remote areas. This implies that poverty may cancel out efficiency benefits a CHC may realize through keeping an intermediate number of horizontal units.

## Conclusion

 Systematic statistical analyses of CHC efficiency are rare, also for the Indonesian context. Using performance information from a sample of 598 Indonesian CHCs, the present study revealed large variations in efficiency, and a clear pattern of conditions causing this variation: both organizational design and context matter for efficiency. With regard to design, horizontal differentiation, but not spatial differentiation, has an impact. With regard to context conditions, *poverty *and *remoteness *indirectly affect CHC efficiency: the efficiency enhancing effect of an intermediate number of horizontal units is exacerbated in non-remote areas, whereas a high proportion of poor people in a service coverage area may temper this effect.

 One of the general objectives of our study was to see to what degree organizational design might matter for improving technical efficiency, and to disentangle potential underlying mechanisms. The fact that we uncovered some significant effects therefore in the first place is theoretically meaningful. Moreover, some of the effect sizes in our analyses are very small, whereas others are sizeable. This holds in particular for the two variables measuring horizontal differentiation.

 This means that CHCs may indeed realize some efficiency gains by changing their organizational design. These gains may be modest and difficult to quantify, but an important policy implication from our study is that some interventions on the organizational design may have non-linear effects. This means that CHC managers need to carefully calibrate such interventions in order to find the optimum point, rather than assuming that improvement can be achieved simply by increasing or decreasing the level of differentiation.

 This conclusion is particularly relevant from a policy perspective. Whereas the socio-economic status of the population in non-remote areas may directly influence CHC efficiency, choosing the right organizational design (ie, an intermediate number of horizontal units) can buffer this effect. CHCs operating in larger service coverage areas are slightly more efficient, though this effect is weak.

 Some limitations to this study have to be taken into account. First, one of the reasons why some relationships do not show up as strongly as predicted relates to the fact that efficiency levels are not observed, but estimated in the first stage. Consequently, the efficiency levels that we use as observations of the dependent variable contain some measurement error. Second, this study is based on cross-sectional data, precluding insights into how efficiency changed over time, eg, due to changes in organizational design. Third, given that the sample of remote CHCs is substantially smaller than the sample of non-remote CHCs might partly explain the lack of statistical significance in the analysis. Finally, we measured CHC input in terms of the number of staff available, not in terms of the actual hours they work. For example, a good nurse in a well-organized CHC in a poor and therefore unhealthier environment would have to help patients almost continuously (because demand is higher), whereas her counterpart in a similar CHC located in a rich area might have less work to do because of a lower demand. These fluctuations in working hours could also explain differences in CHC efficiency, but are not included in the analysis.

 Nevertheless, our findings suggest that the CDP framework is a useful theoretical point of departure for modelling variations in CHC efficiency. Future studies may also benefit from a comparative assessment of high-quality data on the *quality of care* provided by CHCs – a key dimension that the current study could not address.

## Acknowledgements

 The authors would like to thank Mark Huisman for valuable input on earlier versions of this paper. All potential errors remain our own. Suwatin Miharti thanks Bappenas and the National Institute of Public Administration of the Republic of Indonesia for support.

## Ethical issues

 Not applicable because publicly available secondary data was used.

## Competing interests

 Authors declare that they have no competing interests.

## Authors’ contributions

 SM developed the research question, theoretical approach and hypotheses, and the research design. Prepared, cleaned and analyzed the data. Wrote several draft versions and finalized the manuscript. RW contributed to theory development, research design, interpretation of results, and drafting all parts of the manuscript. BL contributed to theory development, research design, interpretation of results, and drafting all parts of the manuscript. Supervised the statistical analysis. LH contributed to developing theory development, research design, interpretation of results, and drafting all parts of the manuscript.

## Disclaimer

 We declare that the views expressed in the submitted article are our own and not an ofﬁcial position of the institution.

## Funding

 The study was funded by a SPIRIT Scholarship grant to Suwatin Miharti. Parts of Rafael Wittek’s contribution took place in the context of the 2017 Gravitation Program *Sustainable Cooperation – Roadmaps to Resilient Societies (SCOOP)*, grant number 024.003.025), funded by the Netherlands Organization for Scientific Research (NWO) and the Dutch Ministry of Education, Culture and Science (OCW). The authors declare that no competing interests resulted from this funding.
